# Progress of Quackery—Hahnemann and Holloway

**Published:** 1847-11

**Authors:** 


*Progress of Quackery—Hahnemann and Holloway.*—The following affords additional insight into the *modus operandi* of the medical cheats called homœopaths, while a contrast is presented by the effects of a rival practice. We always knew, and proclaimed it, that these advocates of a non-medical treatment administer most active remedies when the necessity for them arises:—

*Practices of Homœopathists.*

"SIR,—The account of the substitution of active substances, in anything but infinitesimal doses, for homœopathic globules, &c., as given in the last number but one in the *Gazette*, will not surprise many of those who have been accustomed to investigate closely the practice. I have also adduced analogous instances in the last edition of my work, especially the case of the Duke di Cannizaro, formerly well known in London as Count St. Antonio, who at Milan, being treated homœopathically for some slight ailment, had to take three globules, at intervals of some hours. Having accidentally omitted the morning globule, he said to his valet, when the time for taking the second arrived, that when he ought to take the third dose he would be at the opera, and that being homœopathic globules, it would do no harm to take the three at once, which he accordingly did, and was dead within two hours;—the preparation being one of nux vomica.—Your obedient servant, EDWIN LEE."

"*Case of Death by Holloway's Pills.*

"Mr. Grimwood of Walton, narrates the case of a man, aged 25, long labouring under scrofulous disease of the femur and tibia, by whom he was consulted, on the 25th of June last, for symptoms indicative of inflammation of the intestinal mucous membrane. It appears that the poor man, while in his ordinary state of health, was advised, with the hope of being cured of his old standing complaint, to try Holloway's pills, beginning with five night and morning, gradually increasing the dose to ten, and then recurring to five. These instructions he punctually obeyed, with the effect of producing hypercatharsis, which he endured for about a week, in the belief that unless such an effect were caused, he could not obtain any benefit. This continued, and symptoms of greater severity setting in, Mr. Grimwood's assistance was sought, about ten days or a fortnight after the man commenced taking the pills. He then presented evident symptoms of peritonitis and enteritis in an advanced stage, attended with great exhaustion, from the effects of which he soon sank. A post-mortem examination does not appear to have been made."—*Dublin Medical Press.*

